# Cardiovascular Disease Management in the Context of Global Crisis

**DOI:** 10.3390/ijerph20010689

**Published:** 2022-12-30

**Authors:** Patricia P. Wadowski, Aleksandra Piechota-Polańczyk, Martin Andreas, Christoph W. Kopp

**Affiliations:** 1 Division of Angiology, Department of Internal Medicine II, Medical University of Vienna, 1090 Vienna, Austria; 2 Department of Medical Biotechnology, Faculty of Biophysics, Biochemistry and Biotechnology, Jagiellonian University, 30-387 Cracow, Poland; 3 Department of Cardiac Surgery, Medical University of Vienna, 1090 Vienna, Austria

The outbreak of coronavirus disease 2019 (COVID-19) initiated a pandemic that has deteriorated health care access and thus disadvantaged vulnerable populations [1].

## 1. COVID-19 and Cardiovascular Diseases

COVID-19 is associated with inflammatory and thrombogenic processes affecting the (micro-) vascular system [2,3,4]. Herein, the severe acute respiratory syndrome coronavirus type 2 (SARS-CoV-2) infects endothelial cells, leading to endotheliitis and subsequent endothelial dysfunction [2,3,5].

**Figure 1:**
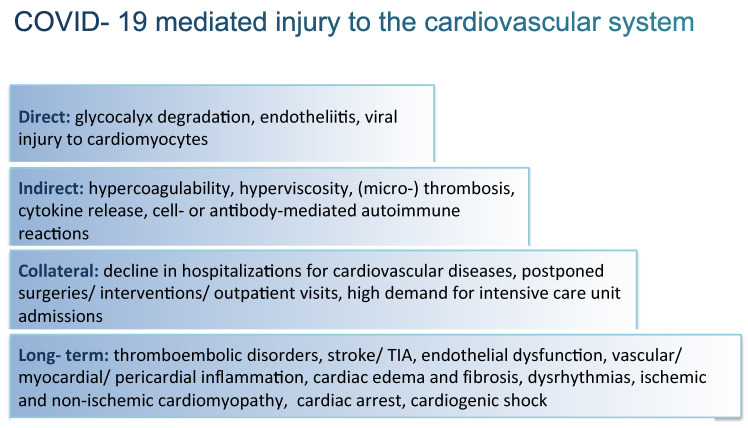
COVID-19 mediated injury to the cardiovascular system.

Microvascular complications due to SARS-CoV-2 infection are especially harmful to patients with preestablished/manifest chronic cardiovascular diseases, wherein capillary perfusion and glycocalyx integrity are disturbed [6,7,8,9,10,11,12]. These patients experience worse outcomes when infected with COVID-19, which is determined by tissue ischemia that, in turn, leads to complications ranging from local infarction to multiorgan failure, limb ischemia, and death [9,13,14]. In the acute phase, direct (viral) and indirect (immune-mediated, pro-coagulatory) myocardial injuries are theorized to contribute to type 2 myocardial infarction and (peri-) myocarditis (Figure 1) [15]. In this context, glycocalyx degradation, endotheliitis, and a burst of inflammatory cytokine release, together with a hypercoagulable state, hyperviscosity, and neutrophil extracellular trap (NET) formation, contribute to the disruption of coronary flow, ventricular dysfunction, and heart failure [15,16,17]. The infection, however, precipitates not only acute but also persistent adverse effects on the cardiovascular system, including the microvascular bed [15,18].

The multilevel damage to the cardiovascular system caused by COVID-19 is primarily conferred by a direct viral infection that mediates glycocalyx degradation and endotheliitis [2,4]. In consequence, multiple pathophysiological processes are induced, such as the activation of platelets and the coagulation system, as well as (auto-) immune reactions contributing to indirect cardiovascular injury [15,19,20,21]. However, the pandemic is accompanied by extensive collateral damage, enhancing the complexity of cardiovascular diseases due to logistic delays in patients’ visits and scheduled treatments [22,23]. Moreover, hospital/intensive care unit admissions due to COVID-19 overburden the capacity of health care systems [24,25]. All these components promote the occurrence and progression of adverse long-term cardiovascular disease, including a procoagulant state with possible intraventricular thrombi [15,26], atherosclerotic plaque destabilization [27], stroke or transient ischemic attack (TIA) [27,28] due to endothelial dysfunction/vasculitis [29]. Furthermore, myocarditis/pericarditis [28], edema [15], dysrhythmias [15,28], cardiomyopathies [27] with late cardiac fibrosis [15], as well as cardiac arrest or cardiogenic shock [28] may occur.

The long-term effects of COVID-19 include—among others—tissue fibrosis, lower left ventricular ejection fraction, and microvascular dysfunction [15,30,31]. The latter was demonstrated by Gao et al., who showed that flow-mediated dilation (FMD) of the brachial artery was significantly lower in patients, even several months after COVID-19 in comparison to the controls [30]. Moreover, the Hamburg City Health Study COVID-19 program showed an increased rate of deep vein thrombosis and the occurrence of atherosclerotic plaque formation in 443 persons after mild or moderate COVID-19 infection within a median of 9.6 months after testing positive for SARS-CoV-2 infection [31].

Endothelial dysfunction is closely associated with platelet reactivity and changes in coagulability during COVID-19 infection [32]. Persisting platelet activation and platelet hyperreactivity after COVID-19 infection may constitute an additional risk factor contributing to post-infectious thrombotic events [26].

Another factor influencing platelet reactivity and worsening cardiovascular diseases is anemia [33,34,35]. The latter may be promoted by SARS-CoV-2 infection, which is thought to interfere with hematopoietic stem cell differentiation, resulting in thrombocytopenia, thrombolytic processes, and acute anemia [36]. Anemia is independently associated with high on-treatment residual platelet reactivity (HRPR) and, together with the latter, confers an increased risk of experiencing ischemic and bleeding events, as demonstrated in patients with percutaneous coronary intervention and stent implantation [34]. In diabetic patients with concomitant chronic kidney disease, anemia elevates the risk of myocardial infarction/fatal coronary heart disease, stroke, and all-cause mortality [33]. During SARS-CoV-2 infection, anemia is associated with a more severe disease course and confers an about 70% higher risk of short-term mortality for patients [37,38].

Further investigation is required regarding the long-lasting effects and pathomechanisms of SARS-CoV-2 injury with respect to the microvasculature.

However, patients with cardiovascular diseases are not solely prone to COVID-19-associated health risks, but also logistic prolems leading to SARS-CoV-2 associated collateral cardiovascular damage (Figure 1) [23]. During the waves of this contagion, a decline in hospitalizations for acute coronary syndromes (ACS) and heart failure was observed [23]. The decrease in hospitalizations for ST-elevation myocardial infarction (STEMI) was greater in low–middle-income countries (LMIC) compared to high-income countries (HIC) [23]. Furthermore, the decline in revascularizations during ACS, concomitant with an increase in thrombolysis, was more pronounced in LMIC [23].

Moreover, in-hospital mortality due to STEMI and heart failure increased in LMIC [23].

The rate of hospitalizations also decreased for arrhythmias, despite an increase in the incidence, especially in areas where COVID-19 is highly prevalent [23].

Regarding patients with peripheral artery disease, an increase in emergency admissions with higher rates of limb loss was observed [22].

Postponed scheduled interventions, surgeries, and outpatient visits constitute only a few examples of the new obstacles faced by patients. 

On the other hand, rapid resource allocation to (severe) cases of COVID-19 and prevention measures to avert the spread of the contagion needed to be immediately established, which proved to be time-consuming processes. 

However, despite the burden of the current pandemic, new diagnostic and therapeutic tools/concepts in cardiovascular medicine are raising hopes regarding the improvement in patient outcomes. 

In the following paragraphs, we will discuss some of these novel and paradigm-changing approaches.

## 2. Best Medical Treatments for Peripheral Artery Disease and Chronic Heart Failure

The current guideline-directed combination of antihypertensive, lipid-lowering, and antiplatelet/anticoagulation treatments is associated with improved patient outcomes with respect to cardiovascular diseases [39,40]. Moreover, there is mounting evidence that strict lipid-lowering strategies prolong (amputation-free) patient survival [41].

Further therapeutic advances, especially for patients with peripheral artery disease, were achievable through a low-dose anticoagulation treatment consisting of rivaroxaban (administered 2.5 mg twice daily) in combination with aspirin (administered 100 mg once daily), which led to reduced major adverse cardiovascular and limb-related events [42,43]. Currently, a triple therapy comprising rivaroxaban, aspirin, and clopidogrel for selected patients with peripheral stent implants and at high risk for recurring ischemic events remains at the discretion of the treating physicians [44].

In patients with severe ischemic left ventricular systolic dysfunction, the results of the ‘Revascularization for Ischemic Ventricular Dysfunction’ (REVIVED) trial will revolutionize treatment concepts as they question the reversal of hibernating myocardium by coronary revascularization [45]. In this study, the patients were randomized with respect to undergoing optimal medical therapy (OMT) according to current guidelines or OMT with an additional percutaneous coronary intervention (PCI). Surprisingly, PCI did not confer a benefit regarding hospitalization for heart failure or death from any cause in comparison to OMT alone [45]. 

Moreover, one can speculate that the results were even potentiated by the fact that the recruitment period started in 2013, when guideline-based OMT did not consist of newer, potent drugs such as angiotensin-receptor neprilysin inhibitors (ARNIs) and sodium/glucose cotransporter 2 (SGLT2) inhibitors [45,46]. The latter proved to be beneficial even in terms of heart failure with a preserved ejection fraction; specifically, empagliflozin reduced the combined risk of cardiovascular death or hospitalization for heart failure [47], and dapagliflozin lowered the combined risk of worsening heart failure or cardiovascular death [48].

However, the (current) results of the REVIVED trial do not describe stenosis severity or associations of stenosis with prior ischemic or viability testing [45,49].

While revascularization in (very-) high-risk patients suffering from STEMI and non-ST-elevation myocardial infarction (NSTEMI) clearly confers a survival benefit [50,51], the results of elective PCI—especially when performed without verifying the degree of stenosis by pressure gradient measurements (fractional flow reserve, FFR)—are less convincing with respect to long-term prognosis [52,53].

Furthermore, in the ‘Fractional Flow Reserve Versus Angiography for Multivessel Evaluation’ (FAME) 3 trial, the comparison of FFR-guided angiography/intervention with coronary artery bypass grafting (CABG) in patients with three-vessel coronary artery disease (CAD) presenting in a chronic or acute (NSTEMI) condition showed the superiority of the surgical treatment [54].

In addition to medical therapy, revascularization by CABG also proved to be superior with regard to survival and hospitalizations in comparison to medical therapy alone in the 10-year follow-up of the ‘Surgical Treatment for Ischemic Heart Failure’ (STICH) trial [55]. Furthermore, in guideline-driven therapy of heart failure with reduced ejection fraction and CAD, especially multivessel CAD and diabetes, CABG is still recommended as the first-choice revascularization strategy [46].

Subgroup analyses and a longer follow-up period of the REVIVED trial will yield further insights into optimized treatment strategies for heart failure patients. 

## 3. Advances in Interventional/Surgical Treatment

The management of patients with cardiovascular diseases involves dedicated treatment and continuous patient follow-ups. Despite the progress in endovascular therapy, patients with chronic limb ischemia that require interventional treatment still face complications such as vessel recoil, dissection, and restenosis [56]. Especially in patients with below-the-knee (BTK) atherosclerosis, medial calcification represents an important pathological feature, reaching 60% prevalence and driving complications [57]. Today, individual approaches such as (drug-eluting) balloon percutaneous transluminal angioplasty (PTA) or the use of drug-eluting stents, intravascular lithotripsy, and atherectomy are the treatments of choice; however, these approaches still require improvement [57,58,59,60]. For complex distal atherosclerotic lesions, retrograde approaches, including the pedal–plantar loop technique, can be successfully applied [61]. However, the induction of diffuse restenosis due to iatrogenic endothelial lesions limits the long-term success of endovascular procedures [62]. Recently, the application of resorbable scaffolds has yielded promising results in infrapopliteal interventions with regard to primary patency rates and limb salvage [63,64]. In addition, the percutaneous deep-vein arterialization technique may improve symptoms and wound healing in patients with critical limb ischemia [65]. 

Advances in device therapy have also elicited distinguished treatment options for selected patients. New pacing methods—such as conduction system pacing, which provides a more physiological stimulus via the His–Purkinje system, or leadless pacing, which may constitute a promising, less-invasive technology—are currently under further evaluation [66]. Over the past years, left ventricular assist device (LVAD) implantation has been increasingly implemented as a long-term/destination therapy due to its improved device technology that precipitates less bleeding and fewer ischemic events [46,67]. This therapy is especially recommended for patients who are ineligible for other surgical options, such as heart transplantation [46,68]. 

Surgical techniques are continuously being evaluated; for instance, the Ross procedure, which is an option for aortic valve repair in young patients, has shown excellent long-term results [69,70]. Moreover, mortality remains lower in comparison to biological and mechanical aortic valve replacement and is comparable among a propensity-matched general population [70].

However, patient outcomes are not independent of surgical skills [71]; thus, dedicated teaching and training of future specialists are required. Furthermore, the training of surgeons in (upcoming) interventional valve repair techniques and endoscopic surgery will be essential to managing prospective demands. Accordingly, interventional transcatheter cardiac valve repair not only entails aortic stenosis and, more recently, insufficiency, but also mitral valve repair, which still remains more challenging [72,73,74]. New imaging techniques and computational models providing three-dimensional reconstructions enable dedicated preoperative planning and postoperative follow-ups [75,76]. Moreover, careful, interdisciplinary, case-to-case patient evaluation by a heart team is crucial for proper management, and even more demanding when an intensive care unit’s capacity is exceeded [77].

In conclusion, patients’ health care management remains challenging during times of global pandemic crises and requires interdisciplinary and international collaboration with respect to discovering innovative solutions with which to foster new therapeutic concepts and regain health care access for critically ill patients.
